# Sero-survey of measles virus antibodies among symptomatic children attending Abuja Teaching Hospital, Nigeria

**DOI:** 10.3205/dgkh000375

**Published:** 2021-01-26

**Authors:** Hafeez Aderinsayo Adekola, Idris Nasir Abdullahi, Anthony Uchenna Emeribe, Nafiu Faruku, Leonard Uzairue, Olusoji Matthew Adeyemi Billyrose, Halima Ali Shuwa

**Affiliations:** 1 Department of Microbiology, Olabisi Onabanjo University, Ago-Iwoye, Nigeria; 2 Department of Medical Laboratory Science, Faculty of Allied Health Sciences, Ahmadu Bello University, Zaria, Nigeria; 3 Department of Medical Laboratory Science, Faculty of Allied Medical Sciences, University of Calabar, Calabar, Nigeria; 4 Department of Microbiology, Federal University of Agriculture, Abeokuta, Nigeria; 5 Department of Medical Laboratory Services, University of Abuja Teaching Hospital, Gwagwalada, Abuja, Nigeria; 6 University Health Center, Faculty of Health and Medical Sciences, Federal University, Dutse, Nigeria

**Keywords:** sero-epidemiology, seroprevalence, measles anti-IgM, measles anti-IgG, vaccination, childhood infection, Nigeria

## Abstract

**Background:** Nigeria is one of the countries with a high prevalence of measles outbreak in children under 5 years old, despite a history of vaccination. This study aims to determine the prevalence of anti-measles virus IgM and IgG among children under 5 years attending the University of Abuja Teaching Hospital (UATH), Gwagwalada, FCT Abuja, Nigeria.

**Materials and methods**: Whole blood was collected, centrifuged, and serum anti-IgM and anti-IgG against measles virus was analysed using ELISA. Sociodemographic variables and vaccination history of subjects were obtained by interview-based questionnaires.

**Results:** The overall anti-Measles virus IgG and IgM seroprevalences were 29.2% and 14.6%, respectively. The prevalence of measles IgG was significantly associated with the parent’s employment status (*X^2^*=11.67, p=0.008). However, the prevalence of measles virus IgM was significantly associated with children’s age (*X^2^*=16.62, p=0.002), parents’ employment status and children’s vaccination status (*X^2^* =7.72, p=0.02).

**Conclusion:** A majority of study participants were not immunised against measles, and a significant number of participants had serological evidence of acute measles virus infection. There is a need for more concerted and massive measles vaccination of children.

## Zusammenfassung

**Zielsetzung:** Nigeria ist eines der Länder mit einer hohen Masernprävalenz bei Kindern unter 5 Jahren trotz früherer Impfung. Deshalb sollte die Prävalenz der Masernviren-Antikörper IgM und IgG bei Kindern unter 5 Jahren, die das University of Abuja Teaching Hospital (UATH), Gwagwalada, FCT Abuja, Nigeria, zur Untersuchung konsultierten, ermittelt werden.

**Material und Methode**: Vollblut wurde zentrifugiert und das Serum Anti-IgM und Anti-IgG gegen das Masernvirus mittels ELISA analysiert. Mittels interviewbasierter Fragebögen wurden soziodemographische Variablen und die Impfanamnesen der Probanden erhoben.

**Ergebnisse:** Die Gesamtseroprävalenz der Anti-Masernviren IgG und IgM betrug 29,2% bzw. 14,6%. Die Prävalenz der Masern-IgG-Antikörper war signifikant mit dem Beschäftigungsstatus der Eltern assoziiert (p=0,008). Die Prävalenz den Masernvirus-IgM-Antikörper war signifikant mit dem Alter der Kinder (p=0,002), dem Beschäftigungsstatus der Eltern und dem Impfstatus der Kinder (p=0,02) assoziiert.

**Schlussfolgerung:** Die Mehrheit der Studienteilnehmer war nicht immun gegen Masern, und eine signifikante Anzahl von Teilnehmern hatte serologische Hinweise auf eine akute Masernvirusinfektion. Daher besteht Bedarf an einer konzertierten, massiven Masernimpfung von Kindern.

## Introduction

Measles is a highly contagious viral disease, caused by measles virus (morbillivirus), that was responsible for millions of deaths prior to the introduction of its vaccine [1]. The virus is an airborne pathogen transmitted through inhalation of infected respiratory droplets or secretions [2]. The incubation period of the disease is estimated to be 10 days before the onset of fever and 14 days before the appearance of rash; other clinical signs include cough, coryza, conjunctivitis and Koplik’s spots, which are pathognomonic for measles [2].

Immunity to measles was thought to be life-long, but reports have shown otherwise, although the incidence has reduced drastically since the advent of vaccines, with 21 countries completely eliminating the virus. Individuals with diminished antibodies or secondary immunosuppression may present with modified and atypical measles [1], [3]. Measles virus re-infection can also occur as a result of vaccine failure. This vaccine failure can be classified as primary or secondary, depending on the concentration of high-avidity measles IgG, which should be present following vaccination and can help confirm secondary vaccine failure [4].

Measles, alongside pneumococcal and rotavirus diarrhoea, are leading causes of vaccine-preventable child mortality [5], [6]. In 2018, despite the availability of a safe, effective and routine vaccination program with at least 83% under the age of one year having access to the vaccine, there was still an estimated 140,000 deaths globally [5]. The recommendation for 2 doses of measles-containing vaccine (MCV) is currently y not driven by endemicity. Local epidemiological patterns and coverage of routinely administered vaccine only influence the age for implementing the vaccine schedule. Delayed vaccination extends the period of the risk of disease for the individual child, but under-vaccination/low coverage is what affects herd immunity. Hence, the current indicator for 2 doses MCV is diphtheria-tetanus-pertussis (DTP3) vaccination coverage level [5], [7]. Delayed vaccination weakens herd immunity and increases the probability of outbreaks due to a large gap between the protection from vaccination and the loss of maternal antibodies [7]. Vaccination coverage is an indicator widely used for evaluating the immunisation program [7]. Therefore, information relating to immunisation is essential to assess the probability of an impending outbreak [8].

Among the Sub-Saharan countries, Nigeria annually ranks top among countries with endemic and continuous transmission of measles [9]. In recent years, a high number of measles cases have been reported, and this has raised many questions about the vaccination program in the country [9]. It has also been reported that vaccinated children become infected with measles, and this further questions the potency of the measles vaccine used in the country [9]. 

Measles has been reported to be a significant contributor to child morbidity and mortality, with 114,900 measles deaths worldwide [10]. Measles antibodies can be used to assess vaccination status and recent infection in children using enzyme-linked immunosorbent assay; this can potentially reveal the status of the individual in a relatively short time. Serological surveys can potentially complement post-vaccination campaign coverage evaluation by providing direct information on population immunity within and outside the target age range of the mass vaccination campaign [9], [11].

Measles can continue to pose a threat to the life of children particularly those under five years of age even with the presence of vaccination programs, so there is a need for routine investigation for this vaccine-preventable disease. This will help to direct empirical policies through appropriate healthcare interventions that will prevent or minimise the morbidity and mortality associated with measles. The present study aimed to determine the serostatus of anti-measles virus immunoglobulin among children under 5 years of age with clinical symptoms of measles attending University of Abuja Teaching Hospital, Nigeria.

## Materials and methods

### Study area

This hospital-based cross-sectional study was conducted at the University of Abuja Teaching Hospital (UATH), Gwagwalada, Federal Capital Territory (FCT) Abuja, Nigeria. The hospital is located between latitude 8°55’ and 9°00’N and longitude 7°00’ and 7°05’E. The hospital serves as a referral facility for specialist medical services, which include pediatric infectious diseases.

### Ethical consideration

Ethical clearance was obtained from the human-research ethics committee of the University of Abuja Teaching Hospital, Gwagwalada, FCT Abuja. Written informed consent was obtained from parents of the children. A structured questionnaire was used to collate the sociodemographic data of participants.

### Sample size determination

Using Fisher’s expression for cross-sectional studies, the sample size of 44 was calculated using a previous study that reported 3.1% measles antibody prevalence among children under the age of 5 in Nigeria [11]. However, this was increased to 89 in order to improve the statistical credibility of the study.

### Study population

Between 20^th^ October to 30^th^ November, 2019, children aged ≤5 years, with at least one of the symptoms of measles and whose parents gave consent to participate in the study were randomly recruited into this study. Children with a history of a chronic disorder such as cancer or diabetes mellitus were excluded. Informed consent was given by children’s parents. 

### Sample collection and processing

Two-milliliter blood samples were collected from individual participants using standard venipuncture phlebotomy. The blood was gently dispensed into sterile plain sample containers. The tubes were labelled appropriately with participant’s identification number.

Sera from these blood samples were separated by allowing the blood to clot at room temperature before centrifuging at 2,500 rpm for 10 minutes. Thereafter, it the supernatant was transferred into serum aliquot containers, stored at –20°C, and analysed within 3 days. During these procedures, all types of work-area contamination, spills and aerosols were minimised.

### Detection of measles virus antibodies by ELISA

Tests for anti-measles virus IgM and IgG were conducted using Euroimmun^®^ (Lübeck, Germany). Indirect ELISA was performed, and results were interpreted according to the kit manufacturer’s instruction. This assay was carried out at the Immunology laboratory of UATH. Appropriate internal and external controls (positive and negative) were included in every run.

The test kit contains 12 microtiter strips, each with 8 break-off reagent wells coated with inactivated cell lysates of BSC-1 cells infected with the “Edmonston” strain of measles virus. In the first reaction step, diluted patient samples, calibrators and controls were incubated in the wells. There, anti-measles antibodies bound to the antigens coating the microtiter wells. The wells were then washed to remove any unbound proteins and non-specific antibodies. In a second reaction step, goat anti-human Ig HRP enzyme conjugate was added to each well. The enzyme conjugate bound to any wells that had human Ig binding to the measles antigen. The wells were washed to remove any unbound HRP enzyme conjugate. 3,3,5,5 tetramethylbenzidine (TMB) enzyme substrate was added. If the HRP enzyme was present in the well (positive reaction), the HRP enzyme reacted with the TMB substrate and produced a blue color. After an additional incubation time to allow color development, a stop solution was added. which turns the blue color yellow and inhibits further color development to allow a stable spectrophotometric reading. The test strips were placed in a microplate reader and the optical density of the color was measured. The amount of antigen specific bound antibody was proportional to the color intensity. The Euroimmun anti-measles virus IgM and IgG tests have a specificity of 96.2% and sensitivity of 100%.

### Data collection

A pretested interview-based questionnaire was used to collate sociodemographic variables of subjects and their parents. Medical records and clinical symptoms were collected from children’s hospital charts (through the assistance of attending physicians and nurses).

### Data analysis

All data were analysed using Medcalc software V.19.0.7. Frequency tables were constructed from categorical data. The chi-squared test was used to determine the relationship between the prevalence of anti-measles virus IgM, IgG and subjects’ sociodemographic variables. p-values <0.05 at a confidence interval of 95% were considered statistically significant.

## Results

Eighty-nine samples were tested, and the study population consisted of children aged 5 years and below attending the pediatric outpatient department and clinics of the University of Abuja Teaching Hospital for medical assistance due to ill health. The mean ± SD age of the participants was 2.4±0.98 (range: 1–5 years). The majority were 3 years old (34.8%). 

**Figure 1:**
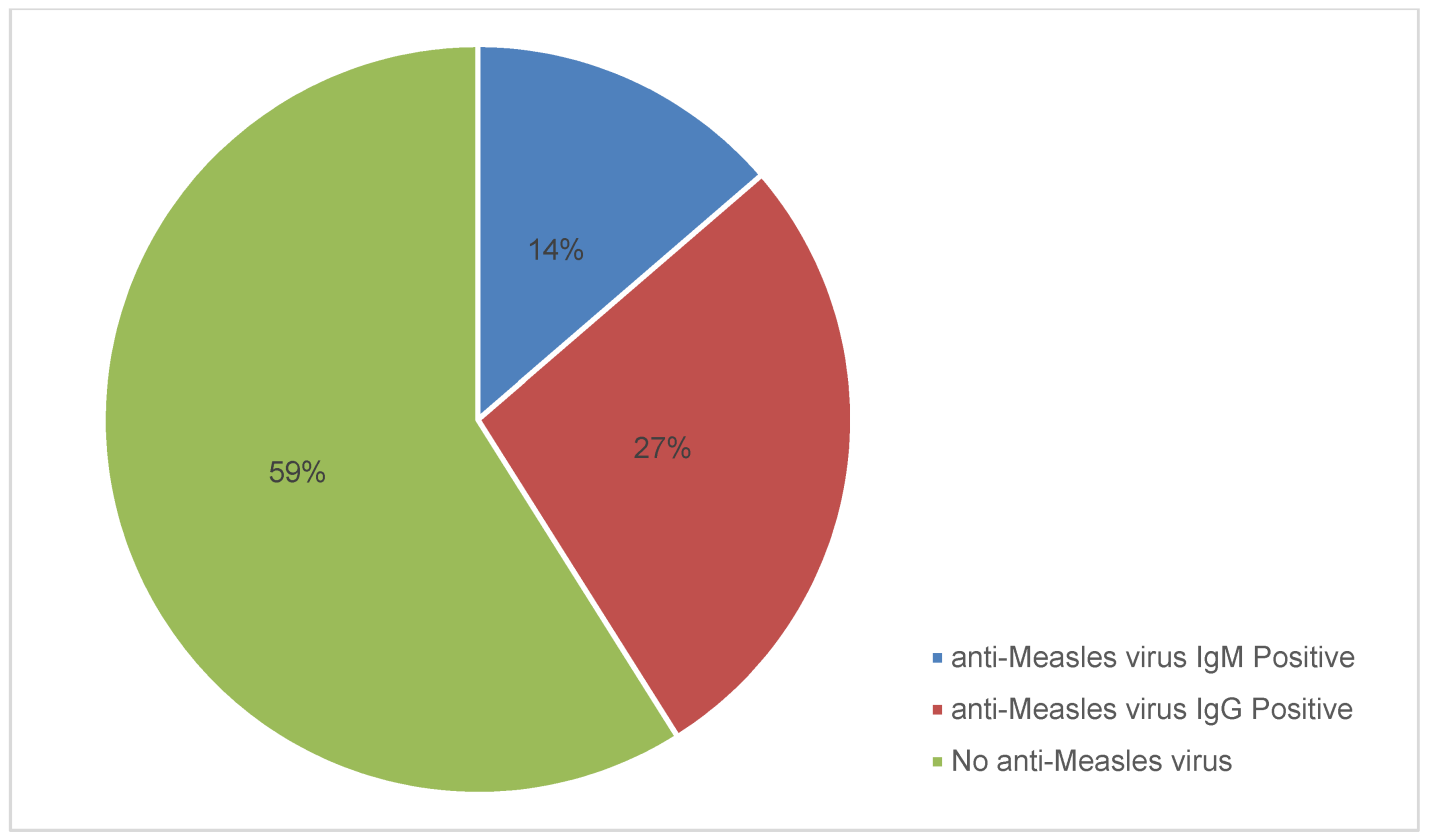
Seroprevalence of anti-measles virus IgM and IgG among children ages ≤5 years

At the first screening, the results of the serological assays were categorised into 4 types of responses. The first group was immune to measles [anti-measles virus IgG (+) and anti-measles virus IgM (–)], and consisted of 20 (22.5%) children. In the second group, 6 (6.7%) children had reactivated measles [anti-measles virus IgG (+) and anti-measles virus IgM (+)]. In the third group, there were 7 (7.9) children who had recent measles [anti-measles virus IgG (–) and anti-measles virus IgM (+)]. In the fourth group, there were 56 (62.9%) children who were susceptible to measles virus infection [anti-measles virus IgG (–) and anti-measles virus IgM (–)] (Table 1 (Tab. 1)). Thus, anti-measles virus IgG seropositivity was 29.2% and anti-measles virus IgM seropositivity was 14.6% (Figure 1 (Fig. 1)).

Girls had a relatively higher prevalence of measles IgG and IgM, 16 (34.8%) and 8 (17.4%), respectively, than did boys, with 10 (23.3%) and 5 (11.6%) cases, respectively. Five-year-old children had the highest prevalence of measles IgG, 4 cases (40.0%). However, 2-year-olds had the highest prevalence of measles IgM, also 4 cases (23.5%). Children from rural areas had a slightly higher prevalence of measles IgG, 13 (29.5%) cases, than did those from urban areas, 13 (28.9%) cases. However, those from urban areas had a relatively higher prevalence of measles IgM (8, 17.8%) than did those from rural areas (5, 11.4%). Children whose parents had no formal education had a slightly higher prevalence of measles IgG (7, 29.3%) than did those whose parents had a formal education (9, 29.0%). However, children whose parents had a formal education had a relatively higher prevalence of measles IgM, 5 (16.1%) cases, than did those whose parents had no formal education, 8 (13.8%) cases. Children of civil servants had the highest prevalence of measles IgG, 6 (40.0%), but children of farmers had the highest prevalence of measles IgM, 7 (21.2%). Children who had had single dose of measles vaccine had the highest prevalence of measles IgG, but no measles IgM. However, children without a history of vaccination had the highest prevalence of measles IgM, 12 (23.5%). The prevalence of measles IgG was significantly associated with the parents’ employment status (p=0.008, *X^2^*=10.32). However, the prevalence of measles IgM was significantly associated with children’s age, parents’ employment status, and vaccination status (p<0.05) (Table 2 (Tab. 2)).

Of the 26 children with measles-IgG seropositive results, 3 (21.4%) only had fever, 9 (30.0%) only had a rash, 2 (25.0%) only had a cough, 4 (50.0%) only had Koplik’s spots, 3 (23.1%) had both fever and rash, 4 (28.6%) had fever and cough, 1 (50.0%) had fever and Koplik’s spots. However, of the 13 children with measles IgM seropositive results, 1 (7.1%) only had fever, 5 (16.7%) only had rash, 1 (12.5%) only had cough, 1 (12.5%) only had Koplik’s spots, 3 (23.1%) had fever and rash, 2 (14.3%) had fever and cough, none had combined fever and Koplik’s spots. The prevalence of measles IgG was significantly associated with children’s clinical presentation (p=0.002, 0.0001, 0.02 respectively) (Table 3 (Tab. 3)).

## Discussion

Measles usually affects children of under five years of age and non-immunised people of any age [12]. The findings from this study revealed 29.2% positive reactions to measles anti-IgG among subjects, which is below the 95% level of measles vaccination coverage recommended by the WHO [13]. Indeed, the seroprevalence of measles IgG varies by country and region, depending on the extent of vaccination coverage and endemicity of measles virus in the general population. This rate is significantly lower than measles IgG seroprevalence reported in other studies. For instance, measles IgG levels of 89.5%, 98.2%, 87.7% and 91.13% were reported in Germany, United Arab Emirates, Italy, and China, respectively [14], [15], [16], [17].

However, our measles IgG value is quite similar to that found in the seroprevalence study of the measles IgG antibody in 1- to 5-year-old children in Zaria, Northwestern Nigeria, which reported a prevalence of 47.80% [18]. This variation (both local and national) is likely due to differences in individual immune responses, differences in the design of immunisation programs, or both [19], [20].

Our studies also show an increased serum antibody against measles in single-dose immunised children, which is similar to the findings by Abdulfatai et al. [18] and Wang et al. [12]. However, children who received 2 doses of vaccination had lower measles IgG seroprevalence. Multiple-dose vaccination offers more protection against measles than does single-dose vaccination [21]. Thus, vaccination failure in those who had seroconverted was shown to be considerably lower than in children who received multiple doses of measles vaccines [22]. 

Another interesting finding in this study is that increase in measles IgG is directly proportional to increase in age, which is similar to the findings of other studies [17], [23], [24]. This may possibly be due to changes in maturation of the immune system or differences in vaccination schedule or both [19]. However, none of the subjects met the WHO standard of >95% IgG positivity in our study. Overall, routine vaccination was not satisfactory. Therefore, there is a need to adopt more easily realized measures that could improve vaccination coverage as an important way to eliminate measles. There was no significant difference in the prevalence of measles IgG antibodies among subjects with educated vs. uneducated parent in this study. This finding not corroborate with those of previous studies [17], [25]. This could be due to the recent house-to-house campaign and activities to vaccinate children, which tend to reach every child regardless of the educational and/or socioeconomic status of their parents. 

Subjects’ living status showed no significant relation to measles IgG reactivity. However, subjects living in urban areas had lower levels of measles IgG than those from rural areas, but the difference was not significant. Additionally, there was no significant difference between anti-measles antibody levels according to gender. This is also in accordance to what was reported in previous studies [21], [26].

This study revealed a measles IgM seroprevalence of about 14%. This is lower than the high prevalence of 62.4% during a recent measles infection reported in a southwestern Nigerian study [9]. This difference could be that their study was conducted at the zenith of a measles outbreak. Many of the children (23.5%) who tested positive to measles-specific IgM had no history of measles vaccination. In addition, one child who had received a dose of measles vaccination was seropositive for measles IgM. Some studies have shown that infection with measles among vaccinated children is related to vaccine failure. However, we were not able to confirm that in our study, mainly due to insufficient data. Failures of primary and/or secondary vaccinations are usually from vaccine denaturation due to storage failures, insufficient viral dosage, or reduced host immunity [9].

As stated earlier, gender was not significantly associated with measles IgM. However, a significantly greater proportion of girls (17.4%) tested positive for measle antibodies compared boys (11.6%). This is in contrast to previous reports that showed increased infection in female than male patients [9], [27]. This difference could be due to the sample sizes of these studies.

Our data show an increase in measles infections among children up to two years of age, then a decline in the infection is observed in older children up to five years. This agrees with previous findings which reported measles to mainly affect children under the age of five years in Nigeria [28]. This is because children of this age range have higher chances of exposure to the measles virus. 

Children whose parents were farmers had the highest prevalence of measles IgM (21.2%). This could be due to unavailability of basic health care, which includes routine childhood vaccination of rural populations. Seroprevalence of measles virus in relation to vaccinated and non-vaccinated children showed a significant association between measles virus infection and vaccination. Vaccinated children had the lowest seroprevalence, while the non-vaccinated children had the highest seroprevalence. This agrees with the findings of Abdulfatai et al. [18] and Ahamdu et al. [29], and with the policy of measles vaccination at 9 months, based on various studies on seroconversion after measles vaccination at different ages [18], [29]. Vaccination against measles has been shown to protect children from severe measles infections, especially those in endemic regions such as Nigeria, thus decreasing measles-related fatality in these regions [10]. 

## Conclusions

We tested children less than 5 years old who presented with fever, presumably with suspicion of measles. Based on the antibody tests, the majority of the children did not have measles and were also probably not vaccinated, as they were seronegative. About 22% were IgG positive, which signifies either prior measles infection or previous vaccination. It is therefore not surprising that the older children were more likely to have a positive IgG result, as most of them probably had had measles in the past, and most measles infections occur in younger children. Of interest were those who tested both IgG and IgM positive, which suggests either previous vaccination and breakthrough re-infection or a second measles infection. It appears that a 2-dose regimen was better than a 1-dose regimen, as there was a breakthrough infection in at least 1 child who had received a single dose. Worthy of note is that there was a very low seroconversion rate in those who received 2 doses of vaccination. Based on these findings, there is a need for more concerted and massive measles vaccination of children.

## Notes

### Acknowledgement

The authors appreciate the technical and logistic support provided by the staff of the immunology laboratory of the University of Abuja Teaching Hospital, Nigeria.

### Competing interests

The authors declare that they have no competing interests.

## Figures and Tables

**Table 1:**
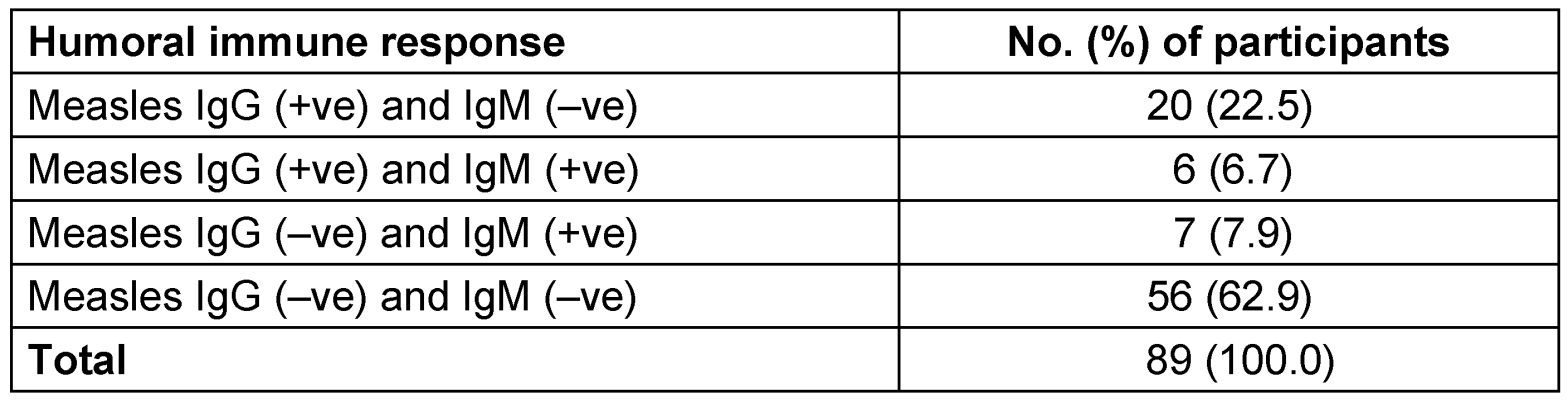
Humoral immune response to measles virus infection among participants

**Table 2:**
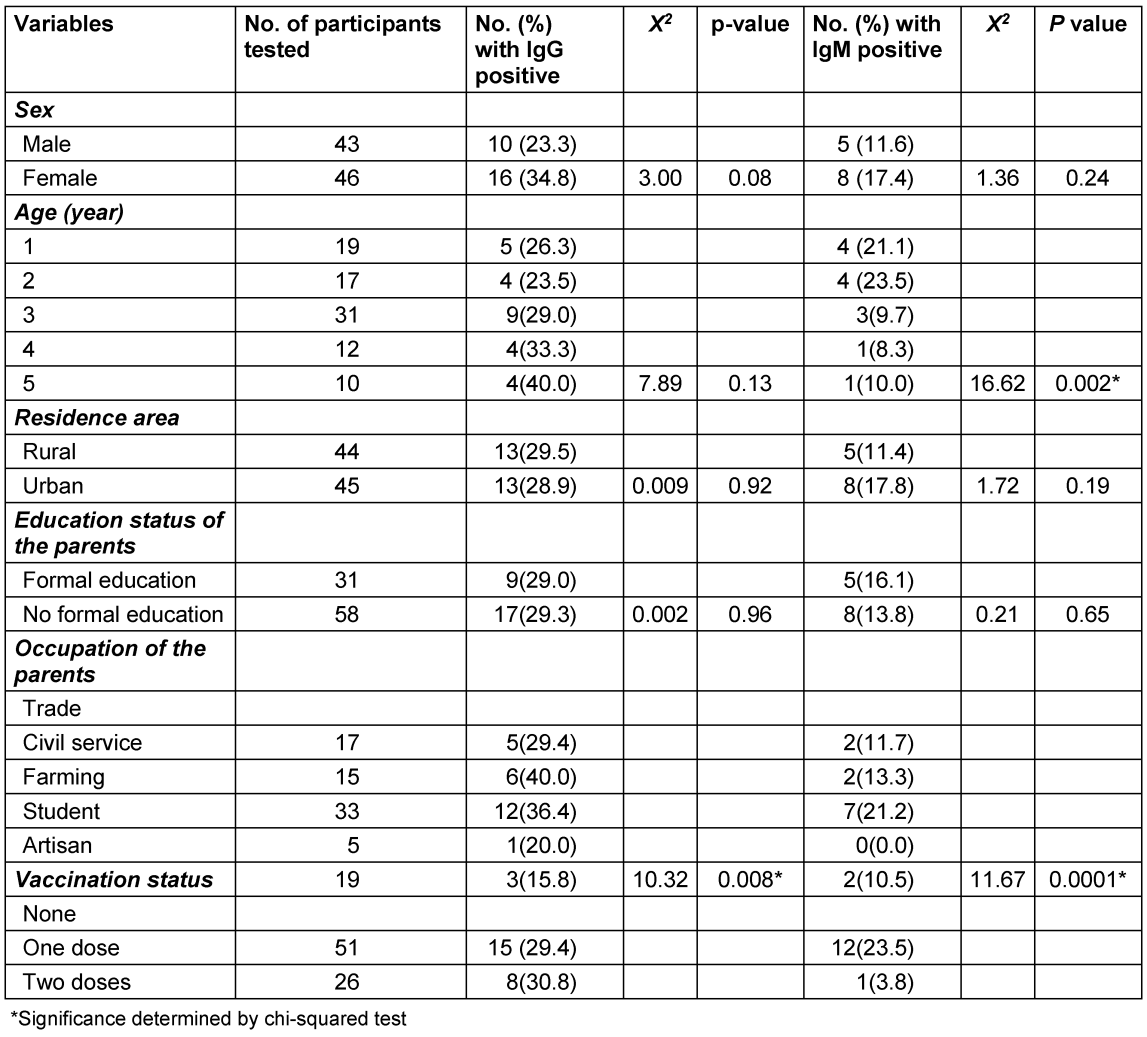
Seroprevalence of measles virus by socio-demographical variables of participants

**Table 3:**
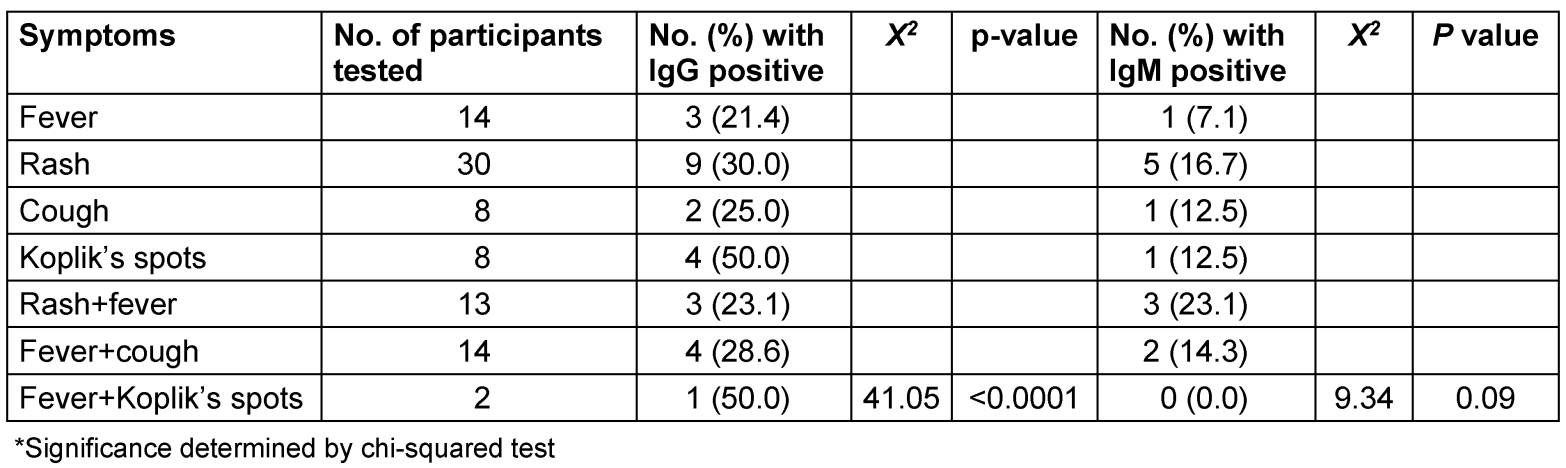
Prevalence of anti-measles virus based on clinical presentations of participants
